# Fronto-parietal network oscillations reveal relationship between working memory capacity and cognitive control

**DOI:** 10.3389/fnhum.2014.00761

**Published:** 2014-09-30

**Authors:** Rasa Gulbinaite, Hedderik van Rijn, Michael X Cohen

**Affiliations:** ^1^Experimental Psychology Department, University of GroningenGroningen, Netherlands; ^2^Department of Psychology, University of AmsterdamAmsterdam, Netherlands

**Keywords:** working memory capacity, cognitive control, fronto-parietal, EEG, theta, connectivity

## Abstract

Executive-attention theory proposes a close relationship between working memory capacity (WMC) and cognitive control abilities. However, conflicting results are documented in the literature, with some studies reporting that individual variations in WMC predict differences in cognitive control and trial-to-trial control adjustments (operationalized as the size of the congruency effect and congruency sequence effects, respectively), while others report no WMC-related differences. We hypothesized that brain network dynamics might be a more sensitive measure of WMC-related differences in cognitive control abilities. Thus, in the present study, we measured human EEG during the Simon task to characterize WMC-related differences in the neural dynamics of conflict processing and adaptation to conflict. Although high- and low-WMC individuals did not differ behaviorally, there were substantial WMC-related differences in theta (4–8 Hz) and delta (1–3 Hz) connectivity in fronto-parietal networks. Group differences in local theta and delta power were relatively less pronounced. These results suggest that the relationship between WMC and cognitive control abilities is more strongly reflected in large-scale oscillatory network dynamics than in spatially localized activity or in behavioral task performance.

## INTRODUCTION

Balancing automatic and controlled behavior is necessary for fast and accurate performance. Insufficient levels of control can lead to errors (Rabbitt and Rodgers, 1977), whereas excessive control slows down responses (Danielmeier and Ullsperger, 2011) or even impairs skilled performance (e.g., performance anxiety; Wulf, 2007). Fluctuations in the levels of control are evident in trial-to-trial changes in reaction time (RT) and accuracy in response-conflict tasks (Eichele et al., 2010), in which task-relevant and task-irrelevant stimulus features prime conflicting responses (Egner, 2008). On congruent trials, in which task-relevant (e.g., color) and task-irrelevant (e.g., location) stimulus features elicit the same response, RTs are faster and responses are more accurate than on incongruent trials, in which task-relevant and task-irrelevant stimulus features call for different responses. This difference is typically referred to as the congruency effect.

The executive-attention theory of working memory capacity (WMC) proposes that high- compared to low-WMC individuals are better at controlling attention, resulting in more stable representations of stimulus-response mappings and less interference from task-irrelevant information (Kane and Engle, 2003; Kane et al., 2007). This theory has received mixed empirical support. For example, although congruency effects can be larger for low- compared to high-WMC individuals (e.g., Kane and Engle, 2003; Weldon et al., 2013), this effect seems to depend on the task and contextual factors such as the ratio of congruent and incongruent trials (Kane and Engle, 2003; Heitz and Engle, 2007; Keye et al., 2009; Morey et al., 2012; Weldon et al., 2013).

**FIGURE 1:**
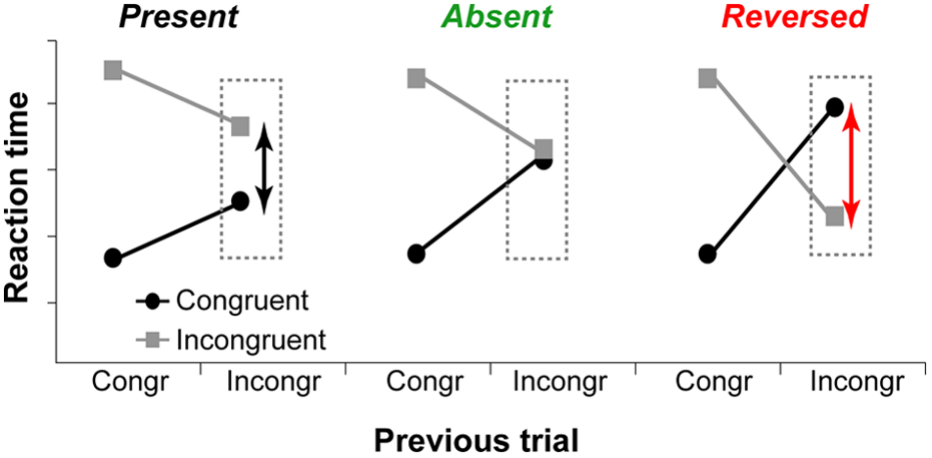
**Hypothetical data show different ways to adapt to previous trial conflict.** Left graph: The influence of task-irrelevant information is reduced compared to post-congruent trials, but the congruency effect is *present*. Middle graph: The influence of task-irrelevant information is minimal or non-existent, and congruency effect is *absent*. Right graph: Task-irrelevant information is actively suppressed, and the congruency effect is *reversed*.

Although the executive-attention theory of WMC does not make specific predictions about WMC-related differences in trial-to-trial adjustments in cognitive control (operationalized as congruency sequence effects), previous studies have demonstrated that differences between high- and low-WMC individuals are more pronounced on post-incongruent trials (Hutchison, 2011; Weldon et al., 2013; Gulbinaite and Johnson, 2014), with modest or no WMC-related differences in post-congruent trial conflict effects. These findings suggest that not only there are WMC-related differences in efficiency of conflict resolution – as proposed by Kane and Engle (2003) – but also differences in how optimally adjustments to the conflict signal are made. Theoretically, as illustrated in **Figure 1**, trial-to-trial adjustments in cognitive control can be: (1) “suboptimal” (influence of task-irrelevant information is moderate, congruency effect after incongruent trials is *present*), (2) “optimal” (the influence of task-irrelevant information is minimal to none, congruency effect is *absent*); or (3) “reactive” (strong active suppression of responses elicited by task-irrelevant information, congruency effect is *reversed*).

In the Simon task, in which incongruence between the task-relevant stimulus feature (e.g., color) and the task-irrelevant feature (location) elicits response conflict, congruency effect on post-incongruent trials is often reversed (responses on incongruent trials are faster than on congruent). Such pattern reflects the fact that task-irrelevant spatial stimulus features always affect performance either by facilitating or by impeding responding. The reversal of the Simon effect has been explained by active suppression of spatially corresponding response, which allows the making of relatively fast responses on incongruent trials, but slows down responding on congruent trials for which response suppression is not needed (Ridderinkhof, 2002; van den Wildenberg et al., 2010).

Reversal of the Simon effect after incongruent trials is larger for low- than for high-WMC individuals (Weldon et al., 2013; Gulbinaite and Johnson, 2014). Following the executive-attention theory of WMC (Engle and Kane, 2004), this pattern of results can be explained by individual differences in the ability to keep task goals continuously active (proactive cognitive control mode). If cognitive control is engaged proactively, the influence of task-irrelevant information is reduced, resulting in a weaker internal conflict signal to which to react (conflict resolution) and to adjust (conflict adaptation). Alternatively, cognitive control processes can be triggered by the stimulus (reactive cognitive control mode), resulting in a stronger internal conflict signal. Studies by Burgess and Braver (2010) and Braver (2012) suggest that a proactive control strategy is more likely to be exercised by those high in fluid intelligence, a measure that is highly correlated with WMC when short-term memory span is partialled out (Conway et al., 2002; de Abreu et al., 2010).

The evidence for the relationship between WMC and cognitive control abilities seems to be highly task-specific, as the relationship between the size of congruency effects and WMC are not always found even in the large-sample behavioral studies (N = 148 in, Keye et al., 2009; N = 189 in, Meier and Kane, 2012; N = 137 in, Keye et al., 2013; N = 262 in, Wilhelm et al., 2013). On the other hand, EEG signatures of response selection and performance monitoring (e.g., error-related negativity) capture WMC-related differences even when behavioral effects are not significant, and thus might be more sensitive measures to study WMC-related differences in cognitive control compared to behavioral measures (Miller et al., 2012).

Functional magnetic resonance imaging (fMRI) findings suggest that fronto-parietal network connectivity might be relevant for individual differences in both WMC and cognitive control abilities (Edin et al., 2009; Faraco et al., 2011; Cole et al., 2012). However, changes in functional connectivity at behaviorally relevant timescales might be missed by fMRI, and cannot be measured with event-related potentials (Cohen, 2011). In contrast, synchronous oscillations between neuronal ensembles have been proposed to be a mechanism for inter-areal communication (Buzsaki and Draguhn, 2004; Fries, 2005), and can be measured with M/EEG data using time-frequency analysis techniques.

The purpose of the present study was to test whether WMC-related differences in cognitive control would be reflected in oscillatory fronto-parietal network dynamics. We recorded EEG while high and low-WMC individuals (as measured by complex span tasks; Redick et al., 2012) performed a Simon task. We focused on theta (4–8 Hz) oscillatory activity over medial frontal cortex (MFC), which has been associated with cognitive control processes (Hanslmayr et al., 2008; Cavanagh et al., 2009; Nigbur et al., 2011; Cohen and Donner, 2013; Cohen and Ridderinkhof, 2013). Both theta power over MFC and phase synchronization with lateral prefrontal sites has been shown to reflect trial-by-trial cognitive control demands and predict RTs during response-conflict tasks (Cohen and Cavanagh, 2011; Cohen and Donner, 2013; Gulbinaite et al., 2014). Due to the novelty of our approach, we also characterized the basic oscillatory interactions between MFC and parietal areas during the Simon task.

## MATERIALS AND METHODS

### PARTICIPANTS AND WMC SCREENING

Participants were selected from a pool of 618 University of Groningen students who had been tested in the automated versions of the Operation span (OSPAN) and the Symmetry span tasks (Redick et al., 2012) in a separate experimental session at least 5 months prior to the Simon task. Previous studies showed high test–retest reliability of complex span tasks, with correlations between sessions ranging from 0.70 to 0.83 (Klein and Fiss, 1999; Unsworth et al., 2005).

In the OSPAN task (Unsworth et al., 2005), participants were instructed to memorize 75 consonants that were serially presented in lists of 3–7 items. Presentation of each letter was followed by a simple arithmetic problem (e.g., 3 + 5 = ?). Next, a one- or two-digit number was displayed until participants indicated “true” or “false” regarding whether the given number was the answer to the arithmetic problem. In the symmetry span task (Kane et al., 2004), participants attempted to memorize 42 spatial locations of serially presented red squares in a 4 × 4 grid, while judging the vertical symmetry of a pattern made up of black squares presented in an 8 × 8 grid. On each trial, spatial locations and patterns were presented in lists of 2–5 items.

Working memory capacity score for each WMC task was computed using the partial-scoring method (Conway et al., 2005), according to which correctly recalled items are given a partial credit if they are recalled in the correct serial position even if the full list is incompletely recalled. All list lengths were weighted equally and the proportion of correct responses was computed for each list length separately (e.g., 2 of 5 = 0.4, 3 out of 3 = 1.0). Thus obtained proportions were averaged across all lists. Individual WMC scores could range from 0 to 1. The scores between Operation and Symmetry span tests correlated significantly [*r*(616) = 0.39, *p* < 0.001]. This correlation is within the range of previously reported correlations between the two tasks (0.36–0.55; Morey et al., 2012; Redick et al., 2012).

For each individual a composite WMC score was computed by averaging z-transformed scores from both WM tasks. As the goal was to characterize a specific dimension of individual differences rather than to estimate the exact effect size, an extreme group design was used (Yarkoni and Braver, 2010). Participants were invited to an EEG session if a composite WMC score fell in the lower (low-WMC participants) or the upper (high-WMC participants) quartiles of the distribution of composite WMC scores in our database (*N* = 618, Q1 = –0.41, Q3 = 0.60).

The required sample size was determined based on the previous EEG study of Miller et al. (2012) using a Simon task, in which error-related brain activity was compared across high- and low-WMC participant groups. To achieve the recommended 80% statistical power at α = 0.05, and an effect size of 0.593 (computed based on the reported $\eta_{p}^{2}$ = 0.26 in Miller et al., 2012), 14 participants per WMC group would be required (computed using G^∗^Power Version 3.1 ANOVA: Repeated measures, within-between interaction; Faul et al., 2007). We tested 19 high-WMC individuals (z-WMC = 0.97, SD = 0.16) and 20 low-WMC individuals (z-WMC = –1.40, SD = 0.51). Data from three participants were excluded due to movement artifacts, one due to poor performance, and one due to technical problems. Thus, 17 high-WMC (eight females, mean age 21.35, three left handed) and 17 low-WMC (15 females, mean age 21.41, l left handed) were included in the analysis. The two WMC groups were gender-imbalanced. To examine whether this may have influenced the results, we conducted several ANOVAs on the main EEG findings using congruency and gender as factors in the high-WMC group (the effects of gender in the low-WMC could not be examined due to the small number of male participants). None of the tests showed a gender effect (the smallest *p*-value was 0.136), and we have therefore not addressed this issue further. All participants had normal or corrected-to-normal vision. The study was conducted in accordance with the Declaration of Helsinki and approved by the local ethics committee. Informed consent was obtained from all participants.

### TASK

Stimulus presentation and response registration were controlled by custom-written Matlab routines using Psychtoolbox (Brainard, 1997). The stimuli were presented on a 17-inch CRT monitor (1024 × 768, 100 Hz) at approximately 90 cm viewing distance.

**FIGURE 2:**
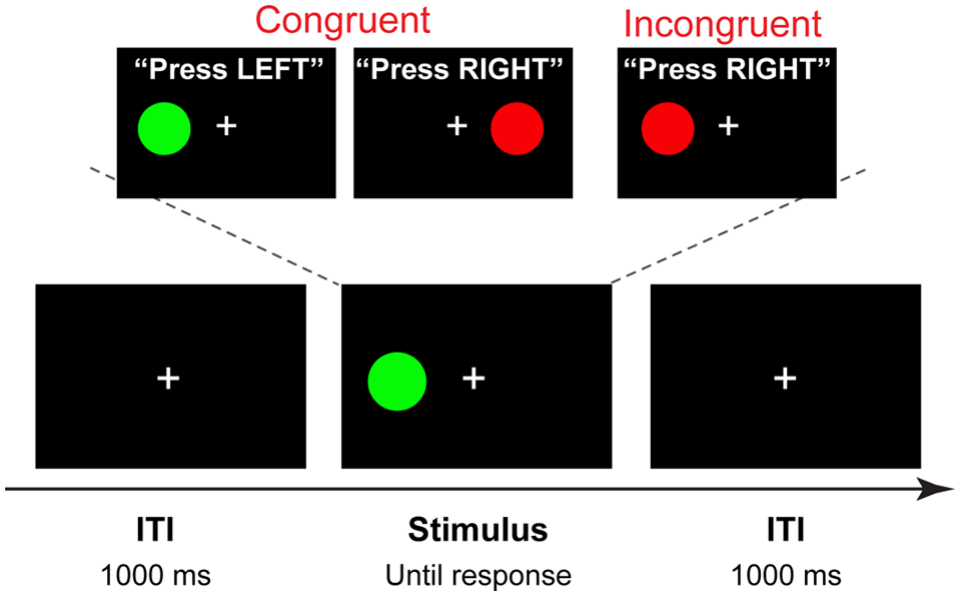
**Trial structure and stimuli.** A four-alternative Simon task, in which two colors are mapped on the left hand, and two on the right hand (counterbalanced across participants). On each trial one of the four stimuli is shown in the location spatially congruent or incongruent with the response hand. In this example, a congruent stimulus is presented with 1000 ms intertrial interval (ITI).

Stimuli for the Simon task were four different color circles, each measuring 2.2 × 2.2 cm (subtending approximately 2& visual angle), presented on a black background 4.5 cm (approximately 5& of visual angle) to the left or right of a white fixation cross. Purple (R: 204 G: 0 B: 204), green (R: 0 G: 104 B: 0), red (R: 204 G: 0 B: 0), and yellow (R: 200 G: 200 B: 0) colors were used, with two stimuli mapped onto each hand. Half of the participants responded to purple and green circle by pressing the “×” key with the left index finger, and to the red and yellow circle by pressing the “>” key with the right index finger; the other half of the participants used the opposite mapping. Each trial began with the presentation of a stimulus to the right or to the left of the fixation cross that remained in view until a response was made or a deadline of 1500 ms was exceeded. After a response was made, a fixation cross was presented for 1000 ms (**Figure 2**).

The overall probabilities of congruent and incongruent trials, trial-to-trial congruency transitions (cC, congruent–congruent; cI, congruent–incongruent; iC, incongruent–congruent; iI, incongruent–incongruent), and the proportions of left- and right-hand responses were kept equal. Due to possible response priming effects on the size of the congruency sequence effects (Mayr et al., 2003), a pseudo-random sequence of stimuli was designed to contain no exact stimulus-response repetitions.

### PROCEDURE

Participants were tested individually in a dimly lit room. They were instructed to respond as quickly as possible while maintaining an accuracy of at least 90%. This was done to avoid ceiling effects in performance and minimize the effect of individual differences in speed-accuracy tradeoff settings. The task consisted of 70 practice trials and 1024 experimental trials. For the first 10 practice trials, feedback on performance accuracy was given after each trial; the remaining 60 practice trials were divided into three blocks of 20 trials each with feedback (mean RT and accuracy) provided after each block. Experimental trials were divided in eight blocks of 64 trials each, with feedback (mean RT and accuracy) provided at the end of each block.

### EEG RECORDING AND PREPROCESSING

Scalp EEG was recorded using 62 tin electrodes (Electro-cap International Inc., Eaton, Ohio, USA) positioned according to a modified version of the international 10-10 system (6 additional electrodes were placed 10% below standard FT7, PO7, O1, FT8, PO8, and O2 electrode positions; F1, F2, CP1, CP2, FT7, and FT8 were not measured). Two additional reference electrodes were placed on the mastoids. Vertical and horizontal eye movements were recorded using four additional electrodes, two of which were placed below and above the left eye and the other two on the outer eye canthi. The data were recorded using the “REFA 8–72” amplifier (Twente Medical Systems, Enschede, The Netherlands), digitally low-pass filtered at 140 Hz and sampled at 500 Hz. All oﬄine data preprocessing and analysis was done using EEGLAB toolbox for Matlab (http://sccn.ucsd.edu/eeglab/) and custom written Matlab scripts (Cohen, 2014).

The data were re-referenced oﬄine to the average activity recorded at the mastoids and high-pass filtered at 0.5 Hz. Continuous EEG recording was epoched from –1500 to 2000 ms around stimulus onset. Trials containing muscle artifacts or eye blinks during the stimulus presentation period were visually identified and removed [on average, 7.97% (SD = 4.35%) of trials per subject]. The second artifact rejection step included independent components analysis (Delorme and Makeig, 2004). Components that did not account for any brain activity, such as eye-movements or noise, were subtracted from the data [on average, 2.29 (SD = 1.32) components per subject]. Furthermore, the first trial of each block, error trials (incorrect or no-response trials), post-error trials, anticipatory responses (RTs faster than 150 ms), and trials in which participants pressed both right and left buttons, were excluded from analyses. Error and post-error trials were excluded to isolate neural processes related to conflict processing and conflict adaptation from error-related processing (Cohen and van Gaal, 2014). The average number of trials per condition included in the statistical analysis for both EEG and behavioral data was: 204 (SD = 18), 182 (SD = 22), 185 (SD = 20), and 199 (SD = 19), for cC, cI, iI, iC trials, respectively.

Artifact-free data were Laplacian transformed prior to analyses. The surface Laplacian is a spatial filter that attenuates low spatial frequencies that can be attributed to volume conduction, and is therefore appropriate for use in connectivity analyses (Winter et al., 2007). Though not a source localization analysis, Laplacian EEG renders the electrodes maximally sensitive to radial sources directly underlying each electrode (Srinivasan et al., 2007). Nonetheless, we report results according to electrode locations rather than putative cortical sources. Topographical locations of the findings are consistent with previous fMRI (e.g., Fan et al., 2003; Liu et al., 2004) and M/EEG source localization (Bialystok et al., 2005; Cohen and Ridderinkhof, 2013; Pastotter et al., 2013) studies of the Simon task.

### EEG TIME-FREQUENCY ANALYSES

Time-frequency decomposition was performed by convolving stimulus-locked single-trial data from all electrodes with complex Morlet wavelets, defined as:

$$e^{i\,2\,\pi \,f\,t}\,e^{-t^{2}/\left(2\,\sigma^{2}\right)},$$

where *t* is time, *f* is frequency which ranged from 1 to 40 Hz in 40 logarithmically spaced steps, and *σ* is the width of each frequency band defined as *n/(2*π*f)*, where *n* is a number of wavelet cycles that varied from 3 to 6 in logarithmically spaced steps to obtain comparable frequency precision at low and high frequencies. Instantaneous power was estimated as the square of the complex convolution signal Z (*power* = real[z(t)]^2^ + imag[z(t)]^2^) and averaged across trials. Power values at each time-frequency point were normalized by converting to the decibel scale to account for power-law scaling of oscillations in different frequency bands (amplitude increases when frequency decreases) by using the formula:

$$10\log_{10}\left(p\,o\,w\,e\,r/b\,a\,s\,e\,l\,i\,n\,e\right),$$

where power from –300 to –100 ms pre-stimulus period served as the frequency band-specific baseline. The phase angle φ_t_ = arctan (imag[z(t)]/real[z(t)]) of the complex convolution result was used to compute frequency-band specific inter-site phase clustering (ISPC), a measure of functional connectivity between the brain areas (Buzsaki and Draguhn, 2004; Fries, 2005). ISPC is defined as trial-average phase angle difference between two electrodes j and k at each time-frequency point:

$$\left|\frac{1}{n}\,\sum_{t=1}^{n}e^{i\,\left(\varphi_{j\,t}-\varphi_{k\,t}\right)}\right|,$$

where n is trial count. Baseline normalization of ISPC values at each time frequency point was performed using percent change transformation: 100(ISPC–*baseline)/baseline*, where *baseline* is the frequency–specific average of ISPC values over –300 to –100 prestimulus time period. Several previous studies have demonstrated that applying the Laplacian to scalp EEG data renders them appropriate for connectivity analyses (Srinivasan et al., 2007; Cohen and Cavanagh, 2011; Nigbur et al., 2012).

### STATISTICAL ANALYSES

Statistical analyses were based on previous research-informed and data-driven approaches. Previous studies have consistently demonstrated that WMC-related differences in cognitive control are driven by differences on post-incongruent trials (smaller conflict effects after incongruent trials; Weldon et al., 2013; Gulbinaite and Johnson, 2014), with modest or no WMC-related differences in post-congruent trial conflict effects. Therefore, we tested WMC-related differences on post-incongruent trials only.

#### Behavioral data

Two sets of ANOVAs were performed. First, the general task effects (collapsing over groups) were evaluated by submitting mean RTs and percentage error to separate repeated-measures ANOVAs with current trial type (congruent and incongruent) and previous trial type (congruent and incongruent) as within-subject factors. Second, WMC effects on conflict adaptation were evaluated in another set of mixed ANOVAs with post-incongruent trial type (congruent iC, and incongruent iI) as within-subject factor, and WMC group (high and low) as between-subject factor.

To compare behavioral results of the current study with the results of our previous large-sample study we performed Pearson’s two-tailed correlation tests between WMC scores and post-incongruent conflict effect in the current and in the previous dataset (N = 181; Gulbinaite and Johnson, 2014). Note that in the previous behavioral-only study a two-choice Simon task was used, with all the stimulus parameters identical to present study. We reanalyzed one condition that matched the design of the current study (equal proportions of congruency repetitions, i.e., cC, iI trials, and congruency alternations, i.e., cI, iC trials). Correlations were re-computed using Spearman’s rho, and the pattern of results was the same.

#### EEG data

Previous studies showed early conflict-related modulations of activity in parietal areas, followed by the later occurring modulations in fronto-central areas (Sturmer et al., 2007; Schiff et al., 2011; Cohen and Ridderinkhof, 2013). Based on these findings, we adopted the following procedure. First, we created topographical plots for power in the theta (4–8 Hz) frequency band in early (50–300 ms) and late (300–600 ms) time windows, time-locked to the stimulus onset and averaged over all trials. Second, electrodes that showed the largest change in condition- and group-averaged power in either the early or the late time window were selected. Third, subject- and condition-averaged time-frequency power plots were constructed for these electrodes. Fourth, time-frequency windows with the largest power increase were selected based on visual inspection (marked in **Figures 5–7** as dashed squares in time-frequency plots), and within this window, the subject-specific time-frequency point with maximum power was found. Note that this selection procedure is independent of any WMC group- or condition-specific differences in power, and therefore could not have introduced any biases into the results. Finally, for each subject, the condition-specific power surrounding 100 ms of the peak time-frequency point was used for statistical analyses. This approach was chosen to preserve subject-specific peak frequency activity (Haegens et al., 2014), which may be correlated with WMC (Moran et al., 2010). For ISPC analyses, the same analysis steps were followed. Group-level statistics were performed using the same procedure used for the behavioral data.

## RESULTS

### BEHAVIORAL RESULTS

**FIGURE 3:**
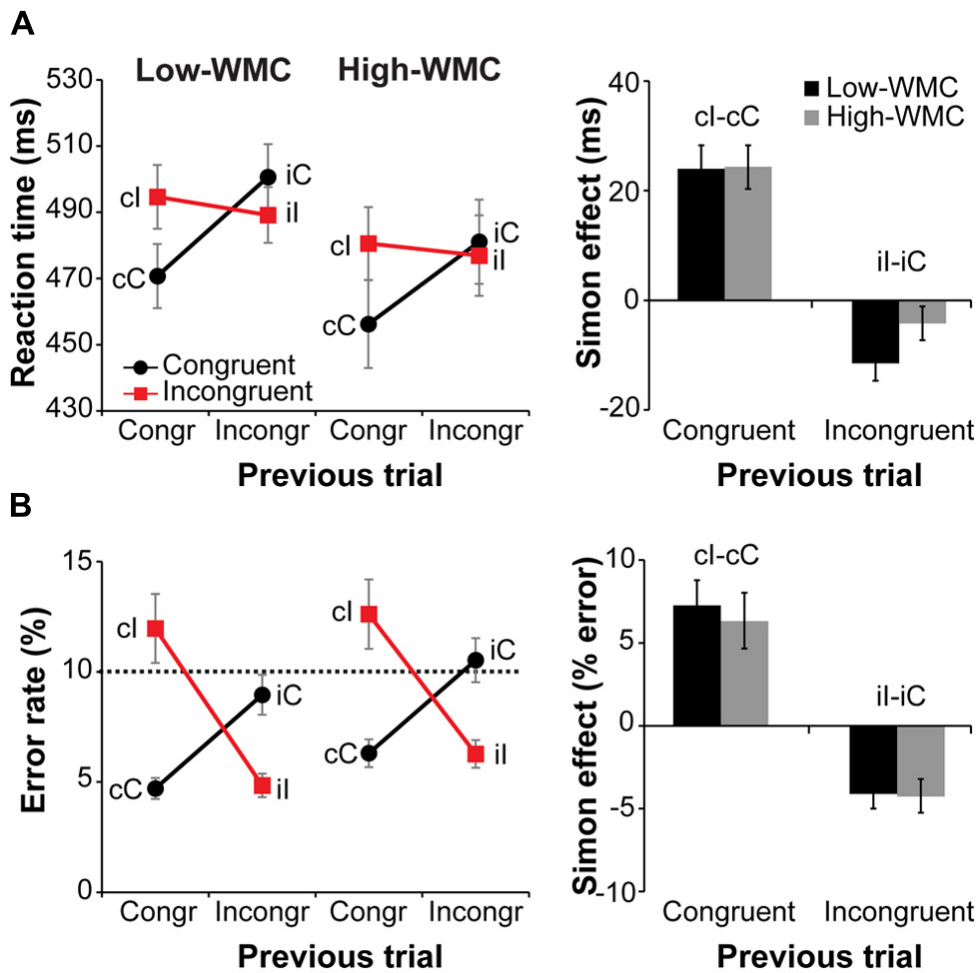
**Behavioral results.** Left-side panels depict reaction times (RTs) **(A)** and error rates **(B)** as a function of current and previous trial type, and working memory capacity (WMC) group. Right-side panels depict post-congruent and post-incongruent trial conflict effects: RT and error rate differences between incongruent and congruent trials. The error bars reflect one SEM. Dashed line in **(B)** denotes 10% error rate (the instructed minimum performance level).

Behavioral results are illustrated in **Figure 3**. Overall RTs on congruent compared to incongruent trials were faster [477 ms vs. 485 ms; *F*(1,32) = 20.81, *p* < 0.001, $\eta_{p}^{2}$ = 0.39] and slightly more accurate [7.6% vs. 8.9% error-rate; *F*(1,32) = 3.54, *p* = 0.069, $\eta_{p}^{2}$ = 0.10]. A current by previous trial type interaction reflected the typical Simon task congruency sequence effects: Positive conflict effect (Simon effect) after congruent trials and a reverse Simon effect after incongruent trials [RTs: *F*(1,32) = 70.36, *p* < 0.001, $\eta_{p}^{2}$ = 0.68; error-rate: *F*(1,32) = 83.76, *p* < 0.001, $\eta_{p}^{2}$ = 0.72; **Figure 3A**, right].

**FIGURE 4:**
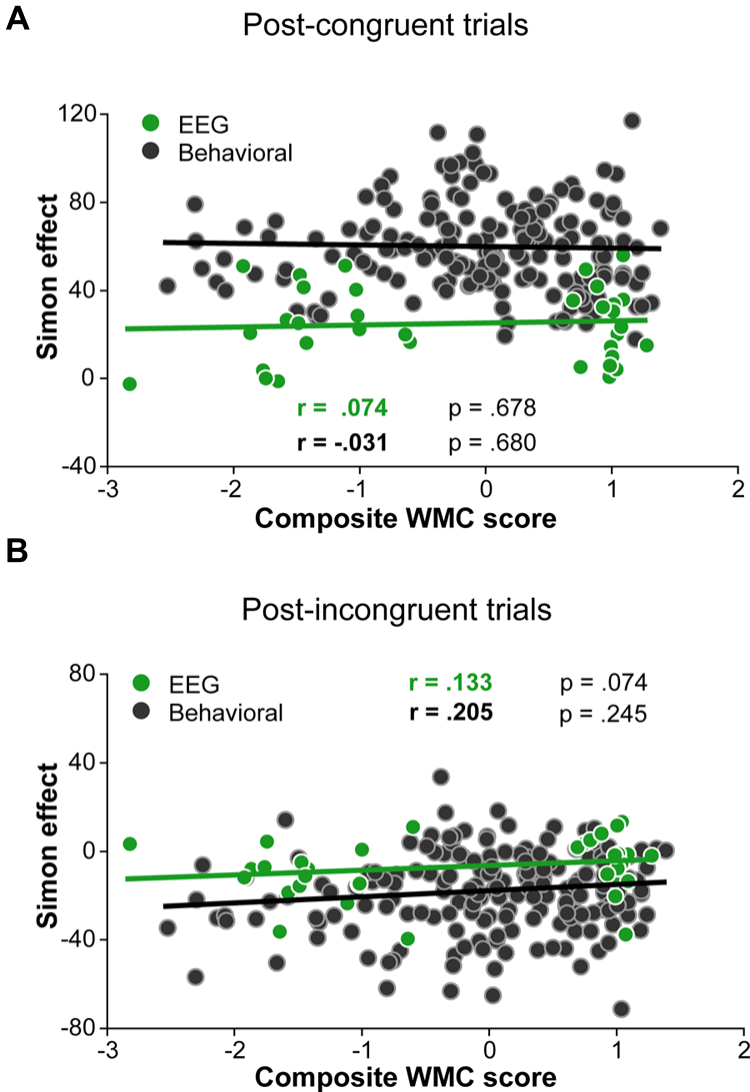
**Pearson correlations derived from 34 participants in the current study and 181 participants in the study by (Gulbinaite and Johnson, 2014). (A)** Correlation between post-congruent trial Simon effect and composite WMC score. **(B)** Correlation between post-incongruent trial Simon effect and composite WMC score.

Although group differences in the conflict effect following incongruent trials were numerically in the predicted direction (larger reverse Simon effect for low- compared to high-WMC group; **Figure 3B**), the WMC x Post-incongruent trial type interaction was not significant [*F*(1,32) = 2.71, *p* = 0.110, $\eta_{p}^{2}$ = 0.08]. To evaluate whether this null finding was a result of small sample size (*N* = 34) in the context of a small effect size, we performed follow-up correlation analyses using data from our previous large-sample study (N = 181; Gulbinaite and Johnson, 2014). Pearson’s two-tailed correlation tests on both datasets were performed (**Figure 4**). The analyses indicated that the relationship between WMC and conflict adjustment was marginally significant in the previous dataset [*r*(179) = 0.133, *p* = 0.074], but failed to reach significance in the current dataset [*r*(32) = 0.205, *p* = 0.245]. According to recommendations by Cohen (1988), the correlation of 0.1 indicates a small effect size. This implies that a large sample size is needed to achieve adequate statistical power and statistically significant results to observe WMC-related differences in behavioral manifestations of conflict adjustment. Specifically, based on the correlation coefficient observed in our previous behavioral study (*r* = 0.133) and that of Weldon et al. (2013; r = 0.22), and an α level of 0.05, 348, and 126 participants (respectively) would be needed to obtain statistical power at the recommended 0.80 level (calculated using G^∗^Power Correlation: Bivariate normal model). The correlation between post-congruent trial conflict effect and WMC did not approach significance in either dataset [*r*(32) = 0.074, *p* = 0.678 and *r*(179) = –0.031, *p* = 0.680].

### EEG RESULTS

**FIGURE 5:**
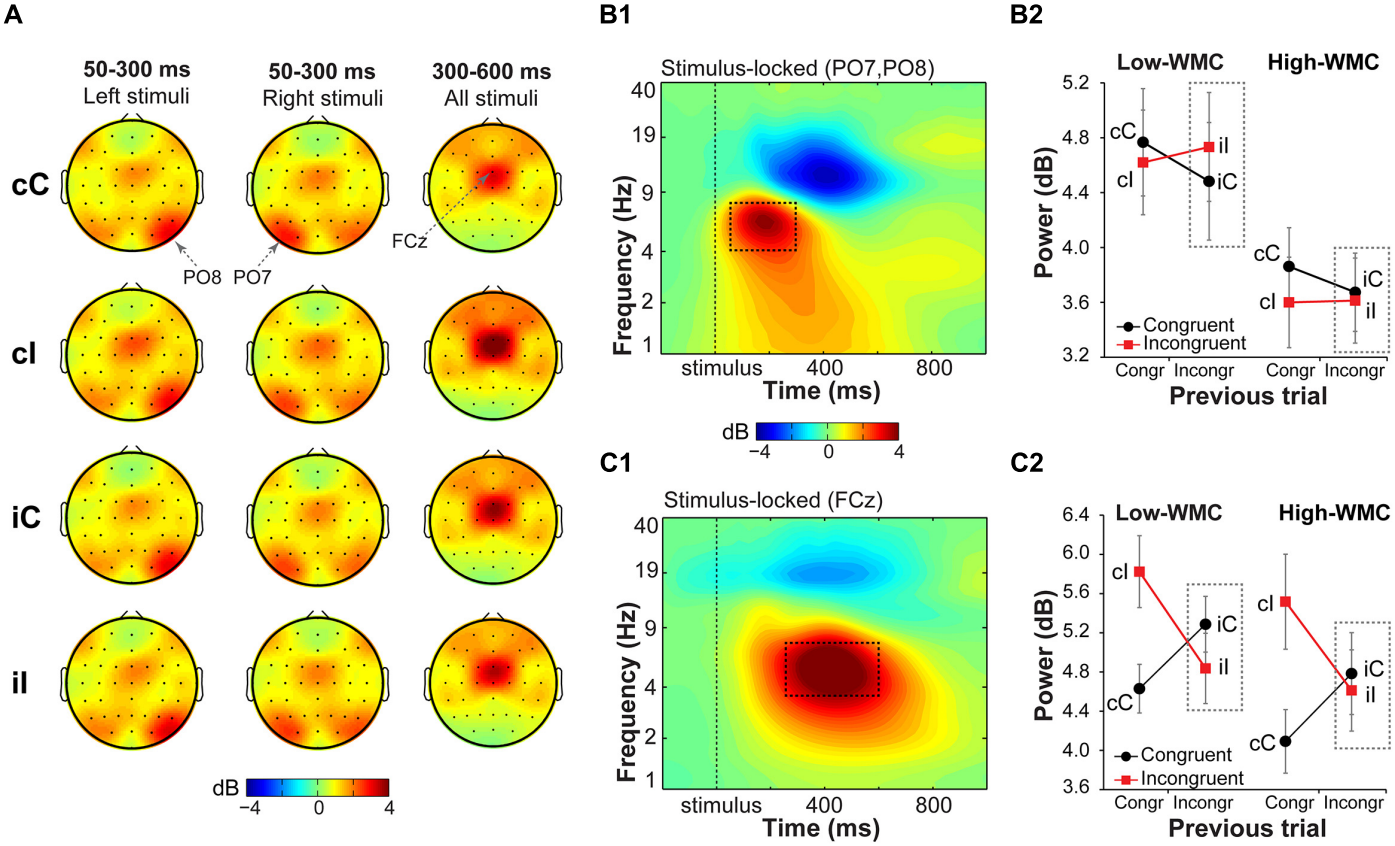
**Task-related changes in theta power. (A)** Topographical maps of power in the theta band (4–8 Hz) averaged over early (50–300 ms) and late (300–600 ms) intervals, separated for previous and current trial type (lowercase and uppercase letters, respectively). Left- and right-hemifield stimulus trials are shown separately to emphasize laterality effects observed over parietal electrodes. **(B1)** Condition-averaged changes in power relative to the baseline (-300 to –100 ms) period over parietal electrodes contralateral to stimulus presentation hemifield (averaged PO8 and PO7); **(C1)** and over medial frontal electrode FCz. Dashed squares represent the time-frequency windows used for the ANOVAs. Condition-specific changes in theta power over parietal **(B2)** and medial frontal areas **(C2)** as a function of previous and current trial type, and WMC group. Dashed squares represent conditions used for WMC-related analyses.

In general, task-related increases in theta-band power compared to the baseline period were observed over stimulus-contralateral posterior parietal areas (spatial peaks around PO8 and PO7, **Figure 5A**) in the earlier time window (50–300 ms post-stimulus), and over midfrontal areas (centered around FCz) in the later time window (300–600 ms post-stimulus; **Figure 5A**). Task-related changes in the delta-band (1–3 Hz; **Figure 7A1**) were pronounced in a 200–600 ms time window over stimulus-contralateral anterior parietal sites (spatial peaks around P3 and P4). We therefore focused our analyses on these time-frequency-electrode regions-of-interest in the power analyses.

#### Parietal theta power

Stimulus-contralateral parietal *theta* power was stronger for congruency repetitions (cC and iI) than for congruency alternations (cI and iC), as indicated by a current and previous trial type interaction [*F*(1,32) = 7.37, *p* = 0.010, $\eta_{p}^{2}$ = 0.18; **Figure 5B2**]. High- and low-WMC groups differed in post-incongruent conflict effects [*F*(1,32) = 4.30, *p* = 0.046, $\eta_{p}^{2}$ = 0.12], such that low-WMC individuals showed a conflict effect [*t*(16) = 3.09, *p* = 0.007], whereas high-WMC individuals did not [*t*(16) = 0.48, *p* = 0.637]. Together these results show that processing of spatial stimulus features in posterior parietal areas was modulated by conflict and by WMC.

#### Midfrontal theta power

Replicating previous findings (Nigbur et al., 2011; Cohen and Ridderinkhof, 2013), incongruent trials as compared to congruent trials elicited a stronger increase in theta power at FCz [*F*(1,32) = 19.23, *p* < 0.001, $\eta_{p}^{2}$ = 0.37]. There was also a significant current and previous trial type interaction [*F*(1,32) = 79.18, *p* < 0.001, $\eta_{p}^{2}$ = 0.71; **Figure 5C2**], reflecting adaptation to the previous trial conflict. However, there were no significant group differences in cognitive control adjustments in response to conflict, as reflected by non-significant WMC and post-incongruent trial type interaction [*F*(1,32) = 1.39, *p* = 0.248, $\eta_{p}^{2}$ = 0.04; **Figure 5C2**].

#### Fronto-parietal theta ISPC

**FIGURE 6:**
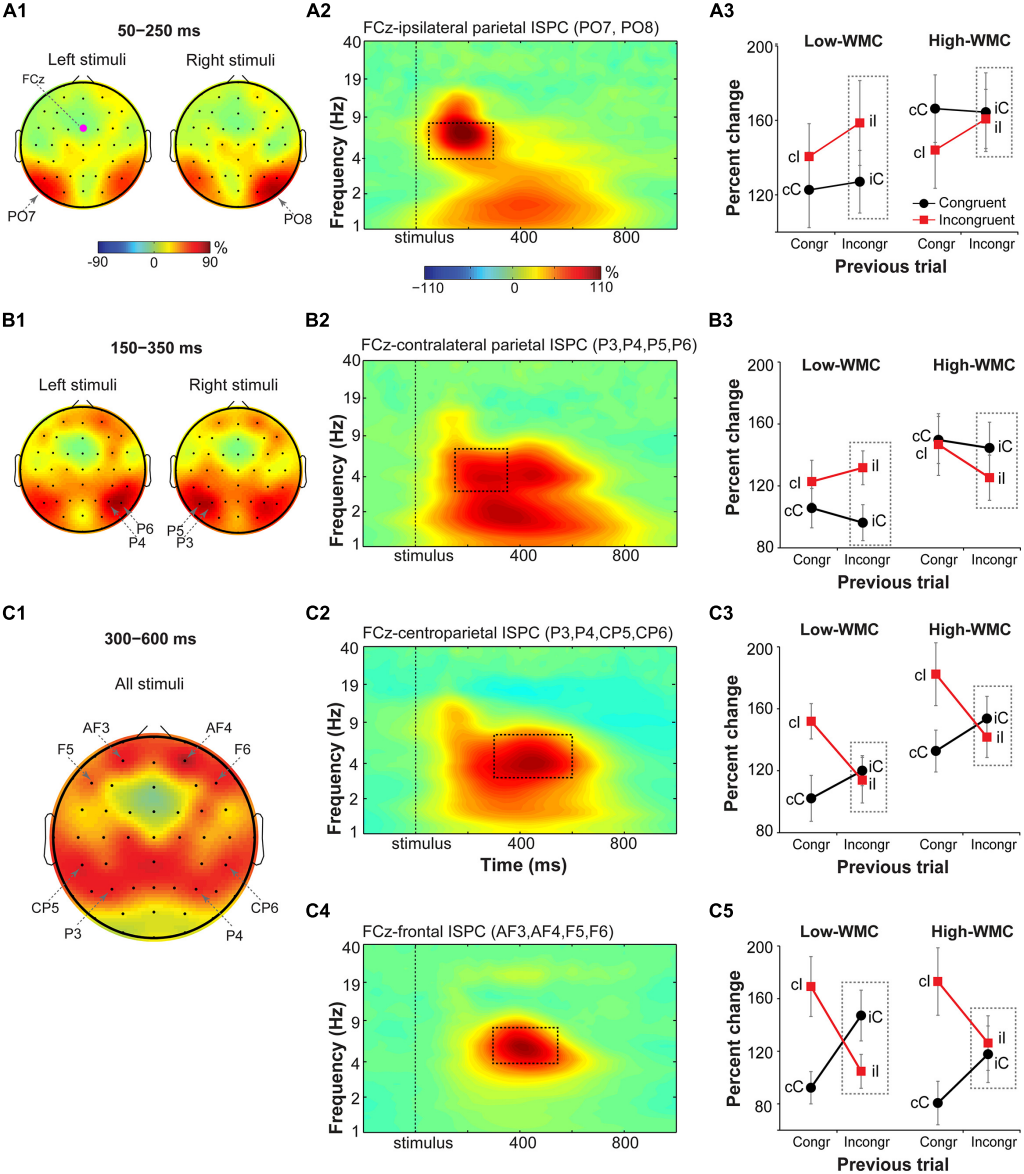
**Task-related changes in theta inter-site phase clustering.** Topographical maps of FCz-seeded ISPC in: **(A1)** early (50–250 ms), **(B1)** later (150–350 ms), and **(C1)** late (300–600 ms) time windows. **(A2,B2,C2,C4)** Condition- and participant-average time-frequency representation of ISPC between FCz (the “seed”) and stimulus-ipsilateral parietal sites (PO7, PO8), stimulus-contralateral parietal sites (P3, P4, P5, P6), bilateral parietal sites (P3, P4, CP5, CP6), and frontal sites (AF3, AF4, F5, and F6). Dashed squares represent the time-frequency windows used for the ANOVAs. **(A3,B3,C3,C5)** Condition-specific changes in theta-band ISPC as a function of previous and current trial type, and WMC group. Dashed squares represent conditions used for WMC-related analyses.

Visual inspection of condition- and group-averaged ISPC data between FCz (the “seed”) and parietal areas revealed increases in theta-band connectivity relative to the baseline in: (1) the early time-frequency window (50–250 ms) over stimulus-ipsilateral posterior parietal sites (spatial peaks around PO7 and PO8 electrodes; **Figure 6A1**), (2) the later time-frequency window (150–350 ms) over stimulus-contralateral anterior parietal sites (spatial peaks around P3, P4, P5, P6 electrodes; **Figure 6B1**), (3) and the late time-frequency window (300–600 ms) in anterior parietal sites bilaterally (spatial peaks around P3, CP5, P4, CP6; **Figure 6C1**). These time-frequency-electrode windows were used as regions-of-interest in the ISPC analyses.

FCz-stimulus-ipsilateral parietal ISPC in the early time-frequency window (50–300 ms; **Figure 6A2**) was not modulated by current or previous trial congruency (*p*’s from 0.182 to 0.283), nor were there group differences on post-incongruent conflict effects [*F*(1,32) = 2.53, *p* = 0.122, $\eta_{p}^{2}$ = 0.07; **Figure 6A3**].

Analysis of FCz-stimulus-contralateral parietal ISPC in the later time-frequency window (150–350 ms; **Figure 6B2**) revealed WMC-related differences in adaptation to the previous trial conflict as indicated by a significant WMC group x Post-incongruent trial type interaction [*F*(1,32) = 7.85, *p* = 0.009, $\eta_{p}^{2}$ = 0.20]. Decomposition of this interaction revealed stronger ISPC on incongruent (iI) vs. congruent (iC) trials for the low-WMC group [*t*(16) = 3.01, *p* = 0.008], with no effect of trial type for the high-WMC group [*t*(16) = 1.24, *p* = 0.235].

Finally, ISPC between FCz and anterior parietal areas in the late time-frequency window (300–600 ms; **Figure 6C2**) was stronger for incongruent than for congruent trials [*F*(1,32) = 9.69, *p* = 0.004, $\eta_{p}^{2}$ = 0.23], replicating similar findings in the Eriksen flanker task (Nigbur et al., 2012). The Current trial type x Previous trial type interaction was also significant [*F*(1, 32) = 21.69, *p* < 0.001, $\eta_{p}^{2}$ = 0.40], reflecting typical congruency sequence effects (**Figure 6C3**). No other effects or interactions reached criteria for statistical significance.

#### Midfrontal-to-lateral-frontal theta ISPC

Based on visual inspection, ISPC between FCz and lateral prefrontal sites (electrodes AF3, AF4, F6, and F5) was evaluated in a 300–550 ms time window (**Figure 6C4**). For consistency with the power analyses, statistics were also performed using a 300–600 ms time window; the pattern of results was the same. There was a main effect of current trial type, with stronger connectivity between FCz and lateral prefrontal areas during incongruent vs. congruent trials [*F*(1,32) = 14.37, *p* < 0.001, $\eta_{p}^{2}$ = 0.30]. The significant interaction between current and previous trial type indicated that ISPC between FCz and lateral prefrontal sites was modulated by the level of conflict on the previous trial [*F*(1,32) = 46.29, *p* < 0.001, $\eta_{p}^{2}$ = 0.58].

There was also a significant interaction between WMC group and post-incongruent trial type [*F*(1,32) = 5.13, *p* = 0.03, $\eta_{p}^{2}$ = 0.14]. Follow-up analyses showed that ISPC was stronger on congruent (iC) than on incongruent (iI) trials for the low-WMC group [*t*(16) = 3.58, *p* = 0.002], whereas for the high-WMC group the effect of trial type was not significant [*t*(16) = 0.45, *p* = 0.663; **Figure 6C5**].

Taken together, these ISPC analyses revealed that the configuration of conflict-related fronto-parietal networks shifted over time: First, ISPC was increased between frontal and stimulus-ipsilateral posterior parietal areas, then between frontal and stimulus-contralateral anterior parietal areas, and finally settled into a bilateral broad fronto-parietal configuration (**Figures 6A1–C1**). Post-conflict adaptation effects in these fronto-parietal network activity patterns were different between the WMC groups already during the early stimulus-processing stage (line plots; **Figure 6B3**) and continued into the later response-selection stage (line plots; **Figure 6C5**).

#### Parietal delta power and fronto-parietal ISPC

**FIGURE 7:**
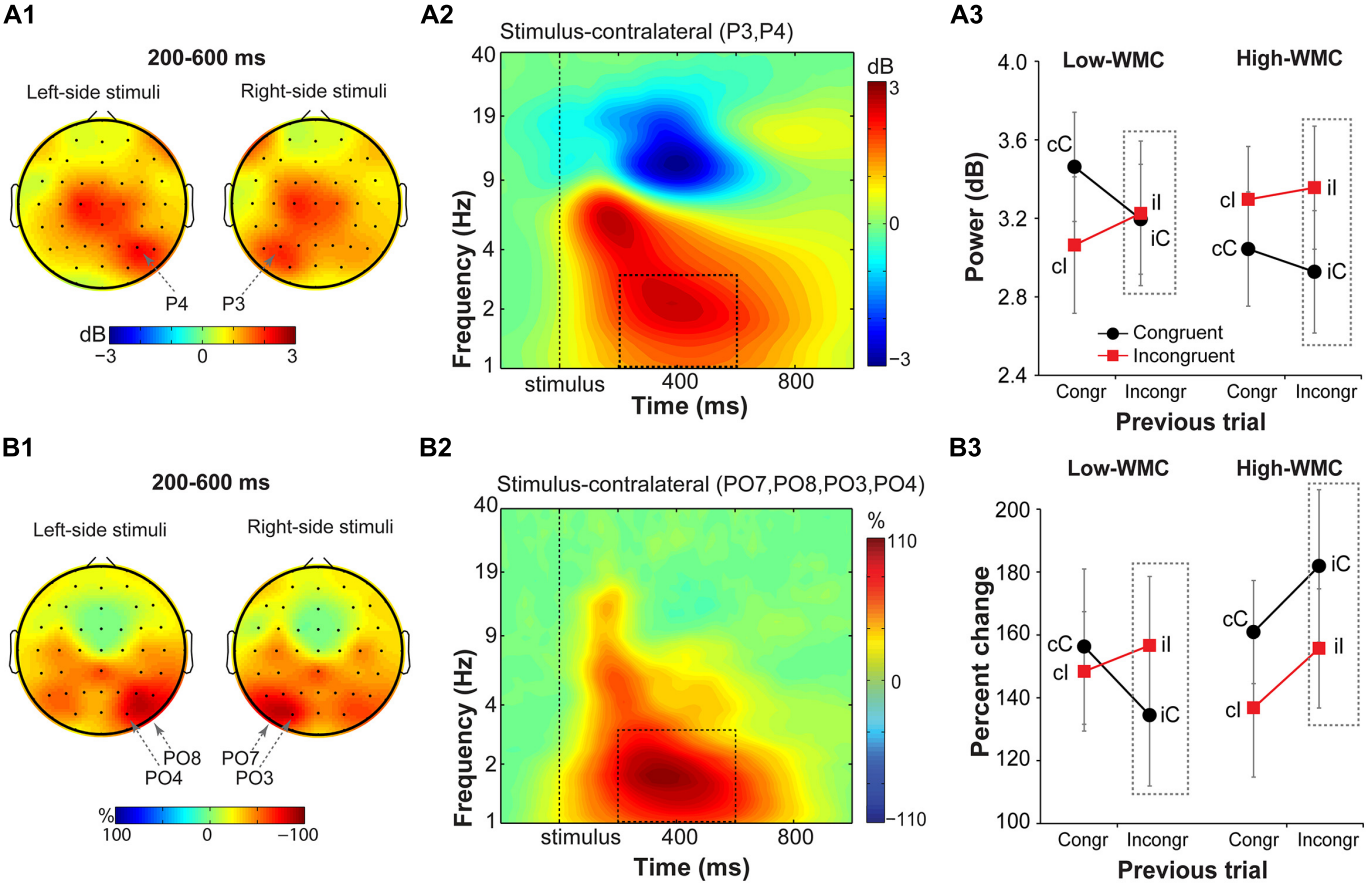
**Task-related changes in delta power and inter-site phase clustering. (A1)** Topographical maps of delta band (1–3 Hz) power and **(B1)** FCz-seeded ISPC averaged over a 200–600 ms time window. Plotted separately for left- and right-hemifield stimulus trials. **(A2,B2)** Time-frequency representation of condition-averaged changes in power and ISPC relative to the baseline period (-300 to -100 ms) over stimulus-contralateral parietal electrodes that showed a maximum peak activity (see **A1,B1**). Dashed squares represent the time-frequency windows used for the ANOVAs. **(A3,B3)** Condition-specific changes in power and ISPC as a function of previous and current trial type, and WMC group. Dashed squares represent conditions used for WMC-related analyses.

Stimulus-contralateral parietal delta power (**Figure 7**) showed a significant current and previous trial type interaction [*F*(1,32) = 4.95, *p* = 0.033, $\eta_{p}^{2}$ = 0.13], with a stronger increase in delta power on congruency repetitions (cC, iI) than on congruency alternations (cI, iC; **Figure 7A3**). There were no group differences on post-incongruent trial conflict effects [*F*(1,32) = 1.38, *p* = 0.25, $\eta_{p}^{2}$ = 0.04].

Analysis of FCz-seeded ISPC revealed increases in stimulus-contralateral posterior parietal electrodes (spatial peaks around PO7, P08, PO4, PO3; **Figure 7B1**). There was a significant WMC group x Post-incongruent trial type interaction [*F*(1,32) = 6.51, *p* = 0.016, $\eta_{p}^{2}$ = 0.17; **Figure 7B3**]. Decomposition of this interaction showed stronger ISPC on iI vs. iC trials for the low-WMC individuals [*t*(16) = 2.22, *p* = 0.041], and no effect of the trial type for the high-WMC individuals [*t*(16) = 1.63, *p* = 0.123].

## DISCUSSION

**FIGURE 8:**
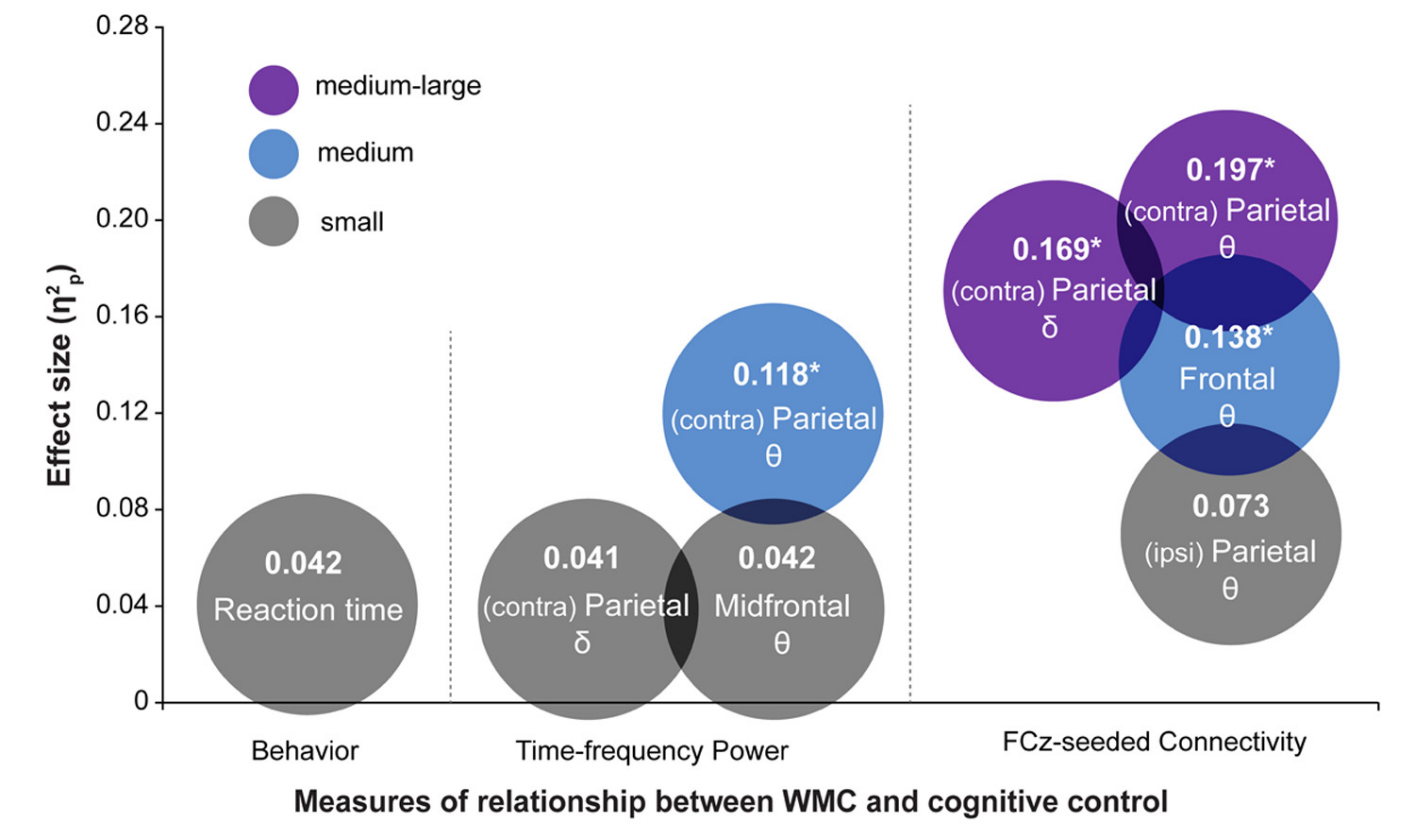
**Summary of results depicting effect sizes of different measures.** Effect sizes are expressed as $\eta_{p}^{2}$. For RT data, $\eta_{p}^{2}$ was calculated as *SS_effect_/(SS_effect_* + *SS_error_)* using results from regression analyses. The color of the circles represents small (gray), medium (blue), and medium-large (purple) effect sizes in the context of the current study. Asterisks indicate analyses in which the effect of WMC was significant. Symbols 𝜃 and δ refer to the results in theta- and delta-band, respectively.

The most striking finding of this study is that the functioning of large-scale networks grouped by oscillatory phase synchronization in theta and delta frequency bands are sensitive markers of WMC-related differences in cognitive control, whereas behavioral task performance did not show statistically significant group differences. These results were further corroborated by comparing effect sizes (quantified as $\eta_{p}^{2}$) of WMC-related differences across EEG and behavioral measures, which are summarized in **Figure 8**. The largest effect sizes for connectivity, power, and behavioral measures were 0.20, 0.12, and 0.04, respectively. This implies that task-related changes in fronto-parietal network connectivity are more sensitive in capturing WMC-related differences than measures of behavior or spatially localized brain activity, and thus smaller sample sizes are sufficient to obtain adequate statistical power and statistically significant results.

The findings documented here extend the executive-attention theory of WMC (Engle and Kane, 2004; Kane et al., 2007), and elaborate on the possible neural mechanisms underlying WMC-related differences in cognitive control. Theta-band oscillatory activity previously has been shown to play a central role within fronto-parietal network communication during attention and cognitive control tasks (Green and McDonald, 2008; Cohen and Ridderinkhof, 2013; Pastotter et al., 2013), and has additionally been associated with working memory (Kahana et al., 2001; Hsieh and Ranganath, 2014).

### NOVEL EEG CHARACTERISTICS OF THE SIMON TASK

In addition to replicating the conflict modulation of midfrontal theta (Nigbur et al., 2011; Cohen and Donner, 2013; Cohen and Ridderinkhof, 2013), we also found an increase in theta activity over stimulus-contralateral posterior areas, likely reflecting processing of the spatial stimulus features (Rusconi et al., 2007; Sturmer et al., 2007; Schiff et al., 2011). Of novelty is the modulation of early parietal theta-band power by preceding trial context, suggesting that cognitive control mechanisms affect processing of task-relevant and task-irrelevant stimulus features already during the early stimulus processing stages (Scerif et al., 2006; Appelbaum et al., 2011; Walsh et al., 2011; Pastotter et al., 2013). Opposite to the behavioral results, congruency repetitions were associated with high, and congruency alternations with low parietal theta power, especially in the low-WMC group (**Figure 5B2**). A similar pattern in the BOLD signal in the fusiform face area in the face-word Stroop task (Egner and Hirsch, 2005), and in ERPs over parieto-central areas in the Eriksen flanker task (Wendt et al., 2007).

This pattern of results can be interpreted considering the proportions of stimulus-response transitions (repetitions and alternations) from one trial to the next. In the Simon task, three types of trial sequences are possible: (1) complete repetitions (stimuli and responses from one trial to the next are the same), (2) complete alternations (stimuli and responses are different), and (3) partial repetitions (stimuli or responses are the same). In the current study, in which only partial repetitions and complete alternations were presented, 2/3 of cC and iI trials were complete alternations, whereas only 1/3 of all cI and iC trials were complete alternations. Because on complete alternation trials, stimulus location always changed with respect to the previous trial, an increase in theta power over parietal areas involved in spatial attention can be expected. Moreover, increased theta power over central areas has been previously reported for stimulus alternations compared to stimulus repetitions (Summerfield et al., 2011). Given that our results are similar to two previous findings (Egner and Hirsch, 2005; Wendt et al., 2007), it is clear that posterior modulation by congruency repetition is observed consistently in a variety of cognitive control paradigms and brain measurements. Additional research, however, is necessary to determine the precise contribution of this pattern to conflict task performance.

Theta connectivity revealed task-related shifts in fronto-parietal networks along a posterior-anterior axis: From stimulus-ipsilateral posterior parietal areas (50–250 ms) to stimulus-contralateral anterior parietal areas (150–350 ms), and finally to a broad bilateral fronto-parietal network configuration (300–600 ms; **Figures 6A1–C1**). The early stimulus-ipsilateral increase in fronto-parietal connectivity may reflect a fast stimulus-driven involuntary orienting of attention, whereas the later changes in stimulus-contralateral and bilateral connectivity may reflect voluntary reorienting of spatial attention (Corbetta and Shulman, 2002; Sawaki et al., 2012). Indeed, there are two critical time periods (130–160 ms and 210–240 ms) for spatial attention orienting (Chambers et al., 2004), during which transcranial magnetic stimulation of parietal cortex attenuates or abolishes the Simon effect (Schiff et al., 2011).

Although little is known about attention-related lateralization effects in the theta band (Green and McDonald, 2008; Thorpe et al., 2012), phase synchronization in the alpha band between lower- and higher-level visual regions is increased contralateral to the attended location, whereas alpha amplitude is decreased, reflecting long-range inter-areal communication and inhibitory processes respectively, (Doesburg et al., 2009; Palva and Palva, 2011). Thus, the observed increase in theta power over stimulus-contralateral parietal areas and early theta synchronization between FCz and stimulus-ispilateral parietal areas, followed by later synchronization between FCz and contralateral-parietal areas, seem to reflect functionally distinct processes.

The novel findings of conflict-related modulation of stimulus-contralateral delta-band power and connectivity highlight that conflict-related processes occur in frequencies and brain networks beyond midfrontal theta (Nigbur et al., 2011; Cohen and Donner, 2013; Cohen and Ridderinkhof, 2013; Pastotter et al., 2013). Both delta (waking and sleep; Harmony, 2013) and conflict-related theta oscillations originate from medial frontal regions (Agam et al., 2011; Cohen and Ridderinkhof, 2013). Previously, increased frontal delta-band activity in cognitive control tasks was reported only during errors (Yordanova et al., 2004; Cohen and van Gaal, 2014). Conflict-modulated delta activity in the present study might be related to delta-band synchronization in the dorsal fronto-parietal network during goal-driven (re)orienting of attention (Daitch et al., 2013).

### GROUP DIFFERENCES IN PARIETAL THETA- AND DELTA-BAND ACTIVITY

Before discussing the main findings of the current study, it is important to note that behavioral and neural indices of conflict task performance are characterized by good to excellent test–retest reliability (Clayson and Larson, 2013; Wostmann et al., 2013). Given that high test–retest reliability indicates that a certain measure captures trait-like characteristics (Segalowitz and Barnes, 1993), we consider the relationship between WMC and cognitive control abilities observed in the current study to reflect stable cognitive traits. Nonetheless, situational factors, such as task context and mental state of the participant (e.g., mental fatigue, stress, sleep deprivation) can influence both WMC measures and cognitive control abilities (Ilkowska and Engle, 2010; Braver, 2012).

Consistent with previous reports that kept the proportions of congruent and incongruent trials equal, WMC was related neither to the size of the conflict effect (Keye et al., 2009, 2013; Weldon et al., 2013; Wilhelm et al., 2013; Gulbinaite and Johnson, 2014) nor to the adaptation to the previous trial conflict (Keye et al., 2009, 2013). However, WMC-related differences in cognitive control adjustments to the previous trial conflict were evident in theta/delta functional connectivity in fronto-parietal networks.

Group differences in conflict adaption were apparent early in the trial during processing of to-be-ignored location of the stimulus. Following incongruent trials, stimulus-contralateral posterior parietal power and fronto-parietal connectivity showed a conflict effect (iI > iC) only in the low- but not in the high-WMC group. Increases in theta power over contralateral posterior areas has been suggested to indicate involuntary shifts of attention (Kawasaki and Yamaguchi, 2012; Ahveninen et al., 2013). Applied to our findings, these group differences could indicate that low-WMC individuals actively suppressed the task-irrelevant stimulus location on iC trials, and thus experienced less attentional capture.

Reactivity of the low-WMC participants to the previous trial conflict was further dissociated in the response-selection stage, as reflected by differences in midfrontal-to-lateral-frontal theta-band synchronization. Previously, enhanced and prolonged synchronization between MFC and lateral frontal sites has been observed in high conflict situations (cI trials and errors) and was suggested to reflect increased cognitive control demands (Hanslmayr et al., 2008; Cavanagh et al., 2009; Cohen and Cavanagh, 2011). Here, we observed significantly stronger theta-band synchronization between MFC and lateral frontal sites on iC than iI trials in the low-WMC group, with no differences in the high-WMC group. It appears that iC trials were associated with higher response conflict in the low-WMC group. Given that spatial stimulus information in the Simon task either facilities (on congruent trials) or impedes (on incongruent trials) response selection process, it is likely that low-WMC individuals had difficulty exploiting the facilitatory stimulus location on congruent trials after incongruent trials. This result again indicates that low-WMC individuals are more influenced by the task-irrelevant stimulus location than high-WMC individuals after encountering the conflict on the previous trial. This interpretation is further supported by the delta-band results, which showed that the post-incongruent conflict effect in fronto-parietal ISPC was present only for the low- and not for the high-WMC group. Although the role of delta-band activity in attention control is not well understood (Daitch et al., 2013), weaker fronto-parietal ISPC on iC trials may reflect disrupted reorienting of attention to the spatial stimulus dimension in the low- vs. high WMC group following incongruent trials.

Taken together, these findings suggest that WMC-related differences in conflict-task performance result not only from differences in conflict resolution – as suggested by Kane and Engle (2003) – but also from differences in cognitive control adjustments in response to conflict. More generally, it points to the differences in cognitive flexibility as being a key difference between high- and low-WMC participants. It appears that low-WMC individuals were less prone to use the task-irrelevant (albeit facilitatory) stimulus location on congruent trials after incongruent trials. This is similar to previous work (Kane et al., 2001; Unsworth et al., 2004; Gulbinaite and Johnson, 2014) showing that low-WMC individuals are slower and make more errors when switching from high-conflict to low-conflict trials, particularly when switches are frequent (Gulbinaite and Johnson, 2014).

Our findings indicate that, overall, low-WMC individuals are more reactive to the contextual effects of the previous trial conflict (**Figure 1**, right). This is generally consistent with the idea that low-WMC individuals are more prone to resolve conflict reactively, whereas high-WMC individuals rely more on proactive cognitive control strategies (Braver et al., 2007; Burgess et al., 2011).

Given the relatively strong association between EEG connectivity and WMC as it relates to conflict processing strategies, EEG connectivity might be a fruitful approach for investigating proactive vs. reactive control mechanisms *per se*. It remains an open question whether reactive and proactive cognitive mechanisms are supported by the same neural networks (Braver, 2012; Irlbacher et al., 2014), and whether the structures in MFC are involved exclusively in reactive initiation of cognitive control (Ullsperger and King, 2010) or it could also facilitate utilization of proactive control (Braver, 2012). Therefore, exploiting trait-like preferences for reactive or proactive control (i.e., WMC), in combination with EEG connectivity measures, and measures of MFC morphology – variations in which are related to working memory and conflict task performance (Fornito et al., 2004; Huster et al., 2009, 2014) – might provide novel insights in proactive and reactive control mechanisms.

## CONCLUSION

By using EEG and employing time-frequency analysis techniques, we provide novel neural evidence for the proposed relationship between individual differences in WMC and attentional control (Kane et al., 2007). The parietal theta power and fronto-parietal connectivity indicate that WMC-related differences in attention control occur early in the trial, and are modulated by the previous trial context. Later changes in theta- and delta-band fronto-parietal connectivity further highlighted group differences in flexibility to adjust top-down control in response to the previous trial conflict. These findings reveal that individual differences in cognitive control abilities are related to WMC, and that measures more sensitive than RT and error rates are required to uncover this relationship.

## Conflict of Interest Statement

The authors declare that the research was conducted in the absence of any commercial or financial relationships that could be construed as a potential conflict of interest.
